# Erratum to “Probing Behavior of *Diaphorina citri* (Hemiptera: Liviidae) on Valencia Orange Influenced by Sex, Color, and Size”

**DOI:** 10.1093/jisesa/ieaa045

**Published:** 2020-06-11

Correction of “Ebert T. A. and M. E. Rogers. 2020. Probing Behavior of *Diaphorina citri* (Hemiptera: Liviidae) on Valencia Orange Influenced by Sex, Color, and Size. J. Insect. Sci.”

DOI: 10.1093/jisesa/ieaa016

In the original version of this article, the supplementary material was not available. This material now appears online. The journal regrets this error.

